# Innovation is often unnerving: the door into summer

**DOI:** 10.1186/1756-0381-7-12

**Published:** 2014-07-17

**Authors:** James D Malley, Jason H Moore

**Affiliations:** 1 Center for Information Technology, The National Institutes of Health, Bethesda, MD, USA; 2 Department of Genetics and Institute for Quantitative Biomedical Sciences, The Geisel School of Medicine, Dartmouth College, One Medical Center Dr, Lebanon, NH 03756, USA

## 

Robert Heinlein gave the following background for his story of the same name. “When we were living in Colorado there was snowfall. Our cat wanted to get out of the house so I opened a door for him but he wouldn’t leave. Just kept on crying. He’d seen snow before and I couldn’t understand it. I kept opening other doors for him and he still wouldn’t leave. Then Ginny [his wife] said, ‘Oh, he’s looking for a door into summer.’” Innovation and discovery in science is often like this: elemental but not obvious. It has these two other properties, things it shares with interestingness: does the initial insight identify deep ignorance, and, are we willing to hike for a long time down that trail to validate the results?

A new domain or continent is likely to have been discovered when, standing on the shore or on the first hill, it seems to extend to the horizon, new in every large and tiny respect, each tree, moss, flying thing unknown to us, while likely easily known to others whom we’ve not met. This immediate sense of deep ignorance should be a confirming property of something most interesting. When the mu meson particle was first discovered in 1936, it arrived with amazing and unexpected properties. At a time when particle physics seemed to be a settled topic, it startled the physics community: the (later) Nobel winning physicist I. I. Rabi, was quoted as saying “Who ordered that?” Deep ignorance and surprise can be taken as reliable markers for valuable outcomes and most interesting results.

But confirming and getting to an understanding of the new result—the muon as it came to be known and many other new particles—took years. It wasn’t until the mid-1970s that these finally were organized as the Standard Model, and then was completed only in 2012 by experimental verification of the Higgs boson. So, novelty is curiously most valuable when it has been thoroughly rendered as Standard and this is unavoidably a long arduous process. Discovery is not for the comfortable, requires a league of collaborators, and it might feel, requires all the time in the world. Such is the nature of biomedical research.

These anecdotes are relevant to the science of biological data mining that is often dominated by simple measures of model interestingness such as accuracy, area under the receiver operating characteristic (ROC) curve, or p-value. While important, these measures only imply a fraction of a model’s story, as shown by the extensive review of Geng and Hamilton [1]. Nine different measures of interestingness are summarized. The first is conciseness or parsimony. The second is coverage: does it apply to a broad portion of the data. The third is reliability, measured by the accuracy or error of a classifier. The fourth is peculiarity, which measures how far away a finding is from others. The fifth is diversity, which measures how different are the elements of a model. The sixth is novelty: is the result new? The seventh is surprisingness, as measured by how unexpected the result is based on existing knowledge. The eighth is utility, that is, how useful is the result. The final criterion is actionability, measuring how applicable a result is to a particular domain.

Each of these criteria can be grouped into objective and subjective categories. For example, conciseness, coverage, reliability peculiarity and diversity are all objective measures because they can be computed using an algorithm or mathematical function. On the other hand, novelty, surprisingness, utility and actionability are all subjective and dependent on the experience and knowledge of the particular domain expert.

Biological data mining is about finding interesting patterns in big data. As with the muon, we need to move beyond settled notions that still dominate data mining, such as measures of reliability. It is only one of many signs and signatures that can announce and advance innovation, can identify the new door, the next new continent.
